# Dosimetric analysis of isocentrically shielded volumetric modulated arc therapy for locally recurrent nasopharyngeal cancer

**DOI:** 10.1038/srep25959

**Published:** 2016-05-13

**Authors:** Jia-Yang Lu, Bao-Tian Huang, Lei Xing, Daniel T. Chang, Xun Peng, Liang-Xi Xie, Zhi-Xiong Lin, Mei Li

**Affiliations:** 1 Department of Radiation Oncology, Cancer Hospital of Shantou University Medical College, Shantou, Guangdong, China; 2 Department of Radiation Oncology, Stanford University School of Medicine, Stanford, California, USA

## Abstract

This study aimed to investigate the dosimetric characteristics of an isocentrically shielded RapidArc (IS-RA) technique for treatment of locally recurrent nasopharyngeal cancer (lrNPC). In IS-RA, the isocenter was placed at the center of the pre-irradiated brainstem (BS)/spinal cord (SC) and the jaws were set to shield the BS/SC while ensuring the target coverage during the whole gantry rotation. For fifteen patients, the IS-RA plans were compared with the conventional RapidArc (C-RA) regarding target coverage, organ-at-risk (OAR) sparing and monitor units (MUs). The relationship between the dose reduction of BS/SC and some geometric parameters including the angle extended by the target with respect to the axis of BS/SC (Ang_BSSC), the minimum distance between the target and BS/SC (Dist_Min) and the target volume were evaluated. The IS-RA reduced the BS/SC doses by approximately 1–4 Gy on average over the C-RA, with more MUs. The IS-RA demonstrated similar target coverage and sparing of other OARs except for slightly improved sparing of optic structures. More dose reduction in the isocentric region was observed in the cases with larger Ang_BSSC or smaller Dist_Min. Our results indicated that the IS-RA significantly improves the sparing of BS/SC without compromising dosimetric requirements of other involved structures for lrNPC.

Radiotherapy is the main treatment paradigm for nasopharyngeal carcinoma (NPC)^1^. Though loco-regional control rate of NPC has been improved significantly in the past decade, local recurrence remains a major problem^2^ with an incidence of 10–36%^3^. Re-irradiation with a tumoricidal dose above 60 Gy is commonly used as a main treatment for locally recurrent NPC (lrNPC) patients^4^,^5^. Clinically, an important factor affecting the local control of lrNPC radiotherapy is the dose administered to the target. In the era of 3D conformal radiotherapy (3D CRT), Wang^3^,^6^ reported lrNPC is clearly dose responsive. The 5 year survival rate was 45% when ≥60 Gy was delivered, but no patient survived in the <60 Gy group. Lu *et al*.^7^ reported an excellent local control rate after high dose intensity-modulated radiotherapy (IMRT) of 68–70 Gy for lrNPC. Similarly, a better local control and survival with escalated dose was observed by Li *et al*.^8^. However, delivery of higher-dose radiation is clinically challenging due to the pre-irradiated condition of the surrounding organs at risk (OARs), such as the brainstem (BS) and spinal cord (SC)^9^. Myelitis and BS necrosis, which are rare but devastating, may occur if the doses delivered to BS and SC exceed the tolerance in the management of NPC patients^10^,^11^. The fact that, in most cases, the BS/SC are proximal to the locally recurrent lesion and have reached the threshold doses in the primary treatment course, aggravates the situation and makes it a challenging task to deliver an adequate dose to the target without causing any correlated damage.

Volumetric modulated arc therapy (VMAT) provides a viable solution for re-irradiation of nasopharyngeal carcinoma in clinical practice^12^. Up to this point, however, little effort has been devoted to optimally utilizing the technical capability of VMAT for lrNPC. In this work, a proposed RapidArc (RA) strategy, referred to as “isocentrically shielded RA (IS-RA)”, was investigated for substantially improved dose sparing of the BS/SC while maintaining the target coverage. A detailed planning study was performed to demonstrate the dosimetric benefits of the IS-RA technique.

## Methods

### Ethics Statement

The protocol was approved by the Ethical Commission of the Cancer Hospital of Shantou University Medical College. Because this was not a treatment-based study, our institutional review board waived the need for written informed consent from the participants. The patient information was anonymized and de-identified to protect patient confidentiality. The methods were carried out in accordance with the approved guidelines.

### Patient characteristics

Fifteen lrNPC patients with Stage rT1–rT4, N0-1, M0 were included in this study, staged according to the American Joint Committee on Cancer (AJCC) 7th edition staging system. Eleven were male and four were female, with the median age of 49 (range, 17–70) years. All the patients received radical chemoradiation in the first treatment and the median time to treatment failure was 16 months (range, 13–22 months).

### CT simulation and target/OAR delineation

All of the patients were immobilized in the supine position in a tailor-made thermoplastic cast from head to shoulders. CT scans with intravenous contrast using a 3 mm slice thickness from the head to 2 cm below the sternoclavicular joint were performed by a CT scanner (Philips Brilliance CT Big Bore Oncology Configuration, Cleveland, OH). The CT images were then transferred to the Eclipse (version 10.0) treatment planning system (Varian Medical System, Inc., Palo Alto, CA) for target and OAR delineation and treatment planning.

The gross tumor volume (GTV) included the recurrent primary lesions and positive lymph nodes, which were determined by the CT, MRI, Positron Emission Tomography (PET) and endoscopic findings. Clinical target volume (CTV) encompassing microscopic disease was defined as the GTV plus margins of 8–10 mm, allowing smaller margins close to critical intracranial structures or the SC. Planning target volume (PTV) was generated to account for setup variability and internal motion by adding 3 mm margins to the CTV. The median volume of the PTV (Vol_PTV) was 89 cm^3^ with the range of 38–209 cm^3^.

The OARs, including the SC, BS, lenses, optic nerves, optic chiasm, temporomandibular (T-M) joints, temporal lobes, oral cavity and parotids were contoured. Planning organ-at-risk volumes (PRVs) were created by adding 5 mm margins to the SC and 3 mm margins to the BS, denoted as PRV-SC and PRV-BS, respectively. Normal tissue was defined as the body subtracting the PTV.

### Treatment planning

Three different plans were created for each patient in Eclipse using the three different techniques, IS-RA, conventional RA (C-RA) and RA with the same gantry and collimator angles as those of IS-RA (RA-SGC). 6-MV photon beams from the TrueBeam linear accelerator (Varian Medical System, Inc., Palo Alto, CA) were employed for all the plans. The Progressive Resolution Optimizer (PRO, version 10.0.28) algorithm was used for RA optimization. The prescribed dose to the PTV was 60–69.9 Gy (2.00–2.33 Gy/fraction) administered in 30 fractions. All the treatment plans were normalized to achieve the goal of 95% of the PTVs covered by 100% of the prescription dose, except for 4 advanced cases where compromises were necessary to protect the critical OARs.

The planning goals of the PTVs and OARs used in this dosimetric study are shown in Table 1. D_x_ represents the dose which is reached or exceeded in x of the volume. V_100%_ represents the % volume covered by 100% of the prescription dose. The planning constraints were fine-tuned, balancing the tradeoffs between the lowest dose to the BS/SC and the acceptable PTV coverage.

**Figure 1:**
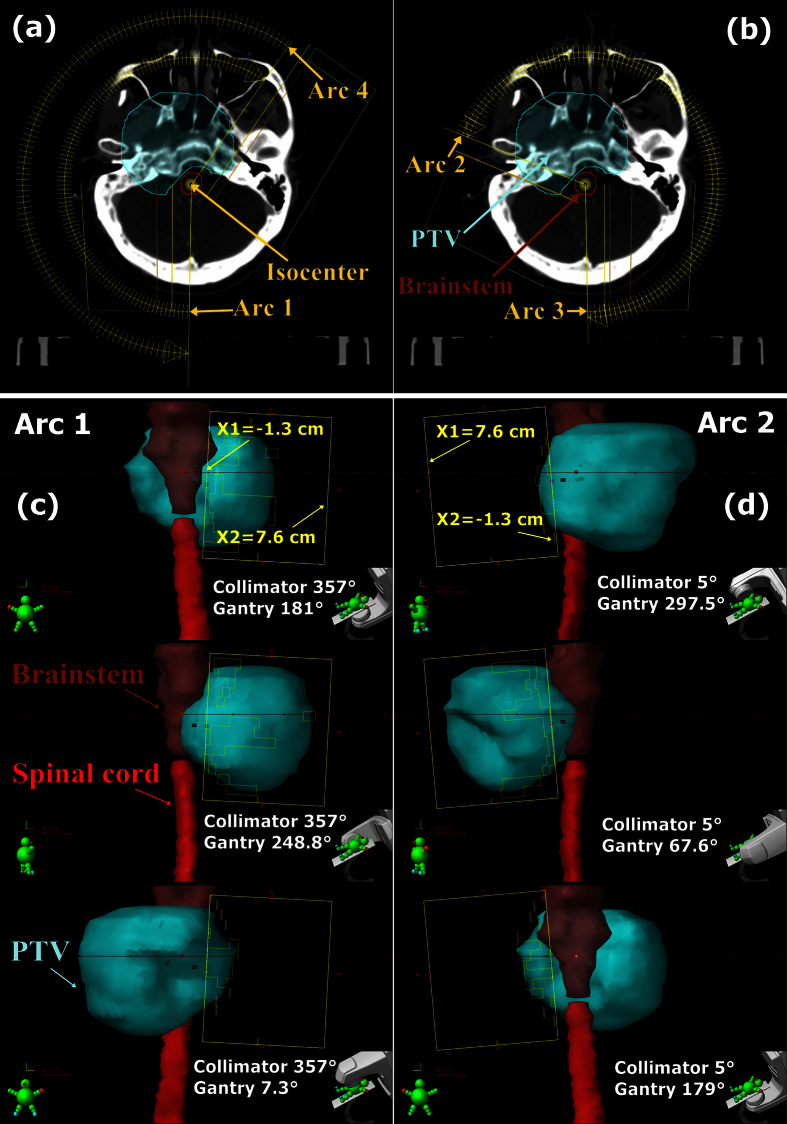
Geometry of the isocentrically shielded RapidArc (IS-RA) technique for a locally recurrent nasopharyngeal cancer (lrNPC) case in Stage rT4N0M0, with the volume of planning target volume (Vol_PTV) of 180 cm^3^, the angle extended by the PTV with respect to the axis of brainstem/spinal cord (Ang_BSSC) of 186° and the minimum distance between PTV and BS/SC (Dist_Min) of 0 mm. (**a,b**) Isocenter location and four partial arcs within two gantry rotations on an axial CT image; (**c,d**) the beam’s eye views (BEVs) in two partial arcs within one gantry rotation.

In generating an IS-RA plan, the isocenter was set at the center of the BS (13 cases) or SC (2 cases), to which the PTV was adjacent. Four or six coplanar partial arcs were placed in two gantry rotations (two or three partial arcs in clockwise gantry rotation, and the other two or three in counter-clockwise rotation). The collimators rotated to angles approximately parallel to the BS and SC during the gantry rotation, generally 1°–20°. The jaw positions were set individually for each partial arc, with the priority of shielding the PRVs of BS and SC, followed by the partial coverage of PTV, but ensuring full PTV coverage if possible during the whole gantry rotation. The geometry settings of a representative IS-RA plan are shown in Fig. 1. There were some overlaps between the adjacent partial arcs in the cases where the PTV was large and more arc length was required to irradiate every portion of the PTV. One partial arc irradiated one side of the PTV near the BS/SC from the beam’s eye view (BEV) while the other partial arc irradiated the opposite side of the PTV. The gantry start and stop angles for each partial arc were individually determined using the BEV. For the cases in which the PTVs were in proximity to the optic structures, two non-coplanar arcs (gantry 45°–90°) with couch 90° were used to bypass the optic structures and deliver sufficient doses to the PTVs.

For each C-RA plan, the isocenter was set at the center of the PTV. Two coplanar full arcs (gantry angle: 181°–179° and 179°–181°) with the same non-coplannar arcs settings as IS-RA were used. The collimator angles were set to 30° aiming at minimizing the tongue and groove effect. The optimization objectives of the C-RA plans were set the same as those of the IS-RA plans.

In order to discriminate the effects of the isocenter placement in the BS/SC and the prolonged arc length with different collimator rotation (1°–20°), the RA-SGC plans were generated, in which the only differences from IS-RA were that the isocenter was placed at the center of the PTV (but not in the BS/SC) and the jaws were automatically set to fit to the whole PTV (but not manually fixed to irradiate the partial PTV). The same optimization objectives were used. All the plans were conducted by a medical physicist.

### Plan evaluation

To compare the three plans, dose-volume statistics, isodose distributions and cumulative dose-volume histograms (DVHs) were calculated. According to the International Commission on Radiation Units and Measurements (ICRU) report 83, D_2%_ and D_98%_ were selected as near-maximal and near-minimal doses for the PTV, respectively. Homogeneity index (HI) was employed to assess the target dose homogeneity^13^:


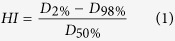


The target dose conformality was measured by the conformity index (CI) introduced by Paddick^14^ accounting for the overlap between the prescription isodose volume (PIV) and the target volume (TV):


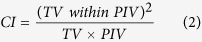


**Figure 2:**
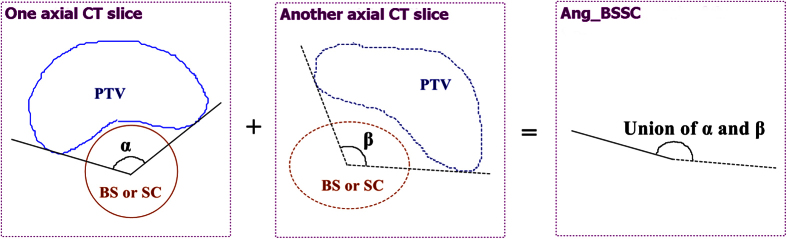
Definition of the angle extended by the PTV with respect to the axis of brainstem/spinal cord (Ang_BSSC).

The CI and HI values were both between 0 and 1, with 1 and 0 indicating the ideal conformity and homogeneity, respectively. D_5%_ was used to evaluate the dose received by the most heavily irradiated 5% volume of the organ^9^,^15^,^16^. The D_2%_, D_5%_ and mean dose (D_mean_) were used for evaluating the doses delivered to the OARs. The geometric parameters of the PTV and its geometric relationship with other structures, including the angle extended by the PTV with respect to the axis of BS/SC (Ang_BSSC) (Fig. 2), the minimum distance between PTV and BS/SC (Dist_Min) and the Vol_PTV were measured and their effects on the BS/SC sparing were investigated. In addition, monitor units (MUs) per fraction were recorded for all plans.

### Statistical analysis

Statistical analyses were performed using the SPSS (version 19.0) software (SPSS, Inc., Chicago, IL). The comparison among the IS-RA, C-RA and RA-SGC plans were tested with two-sided Wilcoxon signed rank test. The effect of the geometric parameters of the PTV (Ang_BSSC, Dist_Min and Vol_PTV) on the BS/SC sparing was investigated using linear regression analysis. *P*-value of <0.05 was considered to be statistically significant.

## Results

A systematic approach referred to as IS-RA was established for the treatment of lrNPC. In most plans (33 out of 45 plans), the V_100%_ of PTV was equal to 95%. In the remaining 4 advanced cases (12 plans), the V_100%_ of PTV was less than 95%, with the lowest value of 88.4%. The doses of all the OARs were limited to the tolerable levels.

### Target coverage

**Figure 3:**
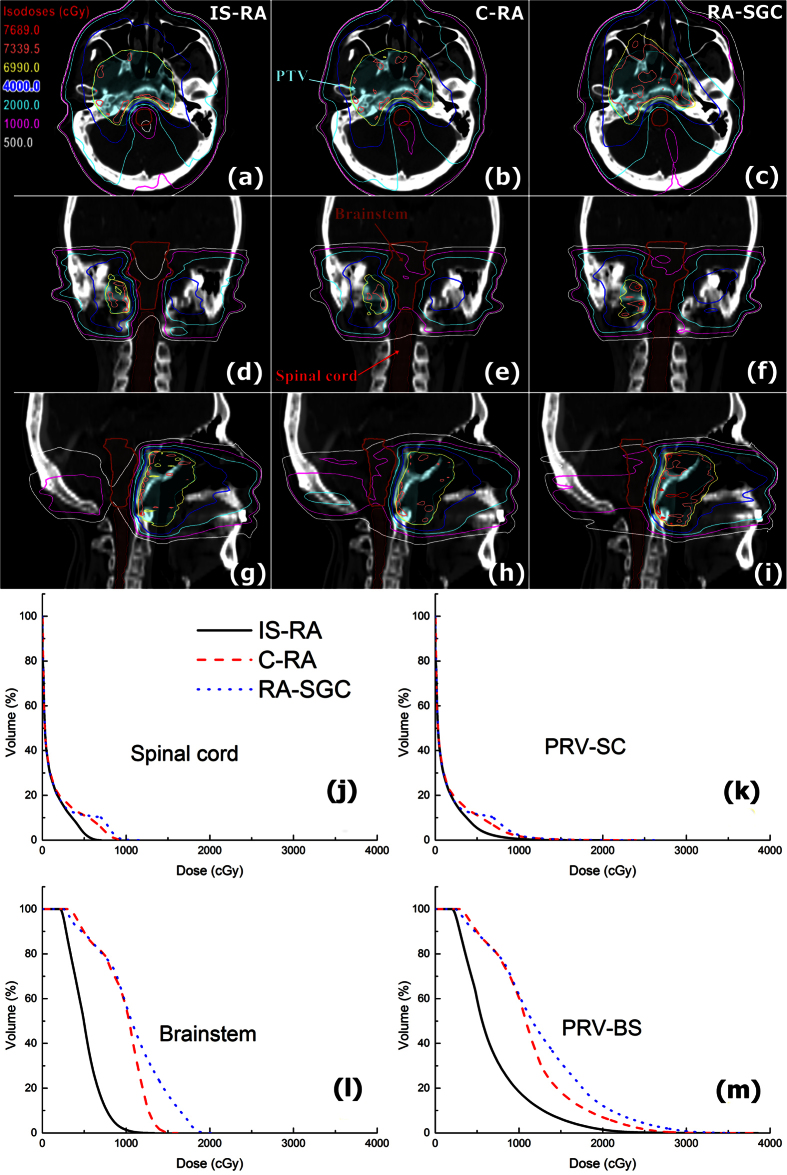
Dose distributions in the axial (**a–c**), coronal (**d–f**) and sagittal views (**g–i**) of the IS-RA, conventional RapidArc (C-RA) and the RapidArc with the same gantry and collimator angles as those of IS-RA (RA-SGC) plans in the same case of Fig. 1; the dose-volume histograms (DVHs) of the (planning organ-at-risk volumes (PRVs) of) BS and SC in this case (**j–m**).

Data of target coverage for all 45 plans is summarized in Table 2. No statistically significant difference was observed with regard to the dose-volume parameters of PTV between the IS-RA and C-RA plans. The RA-SGC plans demonstrated inferior HI, CI and D_2%_. Figure 3 shows the dose distributions of the three plans for a representative case.

### OAR sparing and MUs

The IS-RA spared the BS and SC better than the C-RA and RA-SGC. As shown in Table 2 and Fig. 3, compared to C-RA and RA-SGC, the IS-RA reduced the D_2%_/D_5%_/D_mean_ of the (PRV of) BS by 3.3–3.8 Gy/3.5–3.7 Gy/2.4–2.6 Gy on average. The D_2%_/D_5%_/D_mean_ of the (PRV of) SC was reduced by 2.4–2.8 Gy/2.2–2.5 Gy/0.9–1.1 Gy on average with IS-RA technique. These reductions of BS/SC doses were statistically significant (*P* < 0.05, as indicated in Table 2).

**Figure 4:**
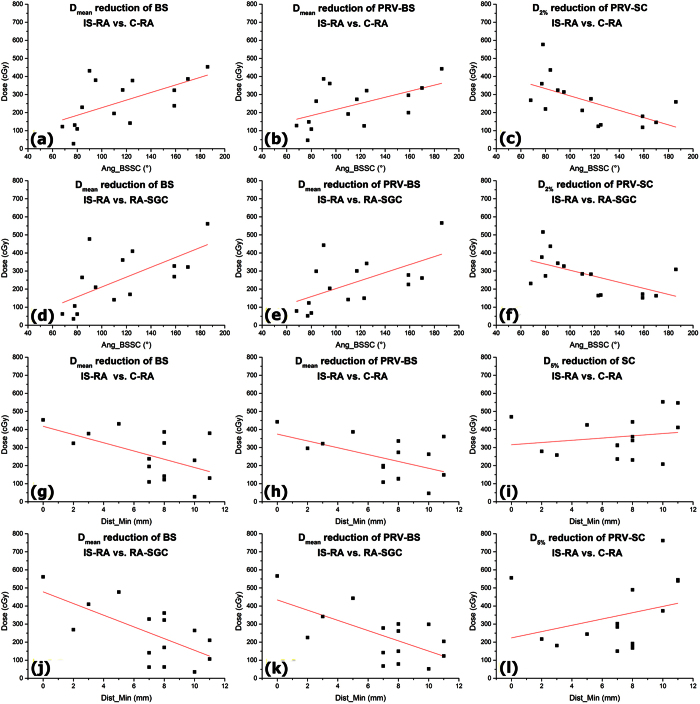
Relationship between the Ang_BSSC (**a–f**)/Dist_Min (**g–i**) and the dose reductions of the (PRVs of) BS and SC by IS-RA when compared to the C-RA or RA-SGC with *P* < 0.05.

The relationship between the Ang_BSSC/Dist_Min/Vol_PTV and the dose reductions of the (PRVs of) BS/SC by IS-RA are demonstrated in Table 3 and Fig. 4, which displays the selected results of geometrical effects with *P* < 0.05. With the Ang_BSSC increasing or the Dist_Min decreasing, the advantage of the proposed IS-RA in sparing the (PRV of) BS became more obvious. However, the level of SC sparing decreased with minor reduction of D_2%_/D_5%_ of the PRV-SC as the Ang_BSSC increased or as the Dist_Min decreased. In the present case (Figs 1, 3 and 4), in which the PTV abut the BS (Dist_Min = 0 mm) with the Ang_BSSC of 186°, the dose of BS was reduced by up to 9 Gy with IS-RA. Besides, Table 3 shows that the Vol_PTV was not a statistically significant impact factor on sparing BS/SC by IS-RA (*P* > 0.05).

It is also noticed that IS-RA exhibited slightly superior sparing of the optic structures, by up to 1.26 Gy in terms of D_2%_ when compared to C-RA. IS-RA also reduced D_mean_ to the normal tissue by up to 0.16 Gy compared to both C-RA and RA-SGC (*P* < 0.05). The doses to most other OARs were comparable among the three plans.

Additionally, the IS-RA produced 40.1 ± 18.9% and 34.9 ± 20.1% more MUs than the C-RA and RA-SGC plans, respectively.

## Discussion

The proposed IS-RA addresses an unmet need in treatment of lrNPC, as it substantially reduces the doses to the BS and SC while adequately covering the PTV. In general, the BS and SC are proximal to the locally recurrent lesions and their threshold doses have often been reached in the primary treatment course, making it challenging to irradiate the recurrent tumor(s) without exceeding the dose limit of the BS and SC. It is technically difficult to reduce even 1-Gy dose to BS/SC, especially in the challenging cases. As compared to C-RA, the IS-RA produces a sharper dose gradient between the PTV and the BS/SC, leading to an improved BS/SC sparing of approximately 1–4 Gy on average (up to 9 Gy in the challenging cases) with simultaneously improved or comparable dose to the PTV and other structures. The efficacy makes IS-RA a useful tool in clinical practice, because reducing the dose to BS and SC to the great extent (ideally 0 Gy) in re-irradiation course may reduce the life-threatening risk of BS necrosis and myelitis. It is noteworthy that the IS-RA technique also reduces the doses to the optic nerves, optic chiasm and lenses, which may be beneficial in reducing the risk of radiation-induced optic complications, such as blindness^17^.

By comparing with RA-SGC, we confirmed that the superior performance of the IS-RA in sparing the BS/SC is mainly attributed to the isocentrically-shielded effect of the OARs with the collimator, not the prolonged arc length or different collimator angles. In geometry, the IS-RA technique focused the beam-shielded projection on the isocentric region during the whole treatment, so the dose of isocentric region could be minimized. During the entire treatment process, the beams across the BS/SC in the isocentric region are avoided, which receives only the scattered radiation from the nearby areas within the open parts of beams^18^ and the negligible transmission through the jaws.

There are a few related techniques for lrNPC that are worth of mentioning. Liu *et al*.^16^ reported that proton beam therapy could achieve a better BS and SC sparing with D_5%_ of 12.83 ± 1.72 Gy and 2.18 ± 1.17 Gy, respectively, while the D_5%_ of BS and SC in photon IMRT were 19.47 ± 1.01 Gy and 13.62 ± 2.17 Gy. The proton beam therapy owes its successful dose sparing to the Bragg peak. However, the proton accelerator is not widely available nowadays. In contrast, the IS-RA technique is capable of producing a comparable D_5%_ of BS and SC using commonly used photon beams, as indicated in Table 2. An IMRT strategy similar to this work was implemented by Chen *et al*.^19^. Using the concept of central block, maximum dose of <15 Gy to the BS and a maximum dose of <10 Gy to the SC were achieved for a prescribed PTV dose of 66–70.2 Gy. The technique, however, required a large number of fields (18–31 fields) and significantly prolonged the treatment time (typically, the delivery took 25–45 minutes). The IS-RA offers similar or even more dosimetric benefits with little overhead in delivery. We also note that Jena *et al*.^20^ proposed a conformal rotation therapy technique with central axis beam block (CRT + BB) for treatment of tumors around the SC. Using the technique, they produced similar SC DVH as compared to IMRT, but at the cost of reduced PTV dose. Cotrutz *et al*.^18^ proposed a technique of intensity-modulated beam delivery that combined the features of the original intensity modulated arc therapy (IMAT) technique and the physical blocking of OAR(s). Lax and Brahme^21^ also utilized a filter to produce a sharp dose gradient between the target volume and the OARs. In those studies, the central “physical block” was applicable only for a cylindrically shaped OAR, typically the SC, but not for the irregularly shaped BS. Furthermore, it takes time to design and fabricate a blocking device. In our IS-RA technique, the collimator jaw is employed for the purpose and thus provides a more practical solution.

One limitation of the proposed technique was the increased MUs, which may increase the risk of secondary cancers in theory due to leakage radiation to patients^22^. Moreover, it should be pointed out that the central axis of the BS/SC is not always parallel to the gantry rotation axis. It is thus difficult to completely shield the BS and SC, which are not regularly and cylindrically shaped, with the shielding jaw during the gantry rotation, leaving a portion of the OARs exposed to the field or only shielded by the multi-leaf collimator (MLC). The further the irradiated area is away from the isocenter, where the center of BS/SC may not be located on the gantry rotation axis, the more likely the BS/SC are incompletely shielded with the jaw. Therefore, as the isocenter was set at the center of BS in our 13 of 15 cases, the dose sparing of the SC was slightly compromised (Fig. 4). For IS-RA treatment of lrNPC, it is recommended that patients be set up in the position in which the axis of the BS and SC are approximately parallel to gantry rotation axis. We note that, the IS-RA lrNPC treatment would be further improved if the jaw position and collimator angle could be optimized for each gantry angle. With the emergence of a new generation of digital linacs, a more general type of treatment planning and delivery techniques referred to as Station Parameter Optimized Radiation Therapy (SPORT)^23^,^24^,^25^,^26^, which optimizes the angular sampling of the control points of VMAT either in coplanar or noncoplanar space, may further improve the dosimetric characteristics of IS-RA in the future.

## Conclusions

The proposed isocentrically shielded RapidArc technique takes advantages of desirable features of rotational arc delivery and central sensitive structure blocking strategy and provides much better sparing of the pre-irradiated brainstem and spinal cord without compromising dosimetric requirements of other organs at risk and the PTV. The technique provides a viable choice for the re-irradiation of locally recurrent nasopharyngeal cancer.

## Additional Information

**How to cite this article**: Lu, J.-Y. *et al*. Dosimetric analysis of isocentrically shielded volumetric modulated arc therapy for locally recurrent nasopharyngeal cancer. *Sci. Rep.* **6**, 25959; doi: 10.1038/srep25959 (2016).

## Figures and Tables

**Table 1:** Planning goals for the IS-RA, C-RA and RA-SGC plans.

**Structure**	**Planning constraint(s)**
PTV	D_95%_ = prescribed dose (60–69.9 Gy)
	D_2%_ < 110% of the prescribed dose
SC	D_2%_ < 10 Gy, D_mean_ as low as possible
PRV-SC	D_2%_ < 15 Gy, D_mean_ as low as possible
BS	D_2%_ < 15 Gy, D_mean_ as low as possible
PRV-BS	D_2%_ < 20 Gy, D_mean_ as low as possible
Lens	D_2%_ < 4 Gy
Optic nerve	D_2%_ < 10 Gy
Optic chiasm	D_2%_ < 10 Gy
T-M joint	D_2%_ < 30 Gy
Temporal lobe	D_mean_ < 12 Gy
Oral cavity	D_mean_ as low as possible
Parotid	D_mean_ as low as possible
Normal tissue	D_mean_ as low as possible

*Abbreviation:* IS-RA = isocentrically shielded RapidArc; C-RA = conventional RapidArc; RA-SGC = conventional RapidArc with the same gantry and collimator angles as those of IS-RA; PTV = planning target volume; SC = spinal cord; PRV-SC = planning organ-at-risk volume of spinal cord; BS = brainstem; PRV-BS = planning organ-at-risk volume of brainstem; D_x%_ = dose which is reached or exceeded in x% of the volume; D_mean_ = mean dose; T-M joint = temporomandibular joint.

**Table 2:** Dosimetric parameters of the IS-RA, C-RA and RA-SGC plans.

	**Parameter**	**IS-RA**	**C-RA**	**RA-SGC**	***P*****-value**		
					**IS-RA vs. C-RA**	**IS-RA vs. RA-SGC**	**C-RA vs. RA-SGC**
PTV	D_2%_ (%)	106.4 ± 1.3	106.5 ± 1.1	107.8 ± 1.4	0.530	0.002	0.001
	D_98%_ (%)	92.4 ± 14.6	92.5 ± 13.3	92.6 ± 12.3	0.307	0.090	0.020
	D_50%_ (%)	103.4 ± 0.9	103.4 ± 0.8	104.8 ± 0.9	0.776	0.001	0.001
	V_100%_ (%)	94.1 ± 1.9	94.1 ± 1.9	94.1 ± 1.9	0.317	0.317	0.317
	HI	0.136 ± 0.144	0.135 ± 0.132	0.144 ± 0.125	0.496	0.027	0.008
	CI	0.818 ± 0.035	0.821 ± 0.032	0.813 ± 0.040	0.191	0.532	0.047
BS	D_2%_ (Gy)	7.45 ± 2.82	11.09 ± 2.04	11.27 ± 2.90	0.001	0.001	0.650
	D_5%_ (Gy)	6.55 ± 2.58	10.14 ± 2.45	10.27 ± 3.29	0.001	0.001	0.955
	D_mean_ (Gy)	3.52 ± 1.39	6.10 ± 2.56	6.03 ± 2.82	0.001	0.001	0.691
PRV-BS	D_2%_ (Gy)	13.26 ± 5.13	16.53 ± 3.95	17.10 ± 4.45	0.001	0.001	0.020
	D_5%_ (Gy)	10.57 ± 4.63	14.02 ± 3.81	14.22 ± 4.40	0.001	0.001	0.532
	D_mean_ (Gy)	4.32 ± 1.88	6.74 ± 2.83	6.67 ± 3.09	0.001	0.001	0.733
SC	D_2%_ (Gy)	4.67 ± 1.74	7.06 ± 1.44	7.16 ± 1.50	0.001	0.001	0.307
	D_5%_ (Gy)	4.24 ± 1.75	6.50 ± 1.67	6.46 ± 1.89	0.001	0.001	0.910
	D_mean_ (Gy)	2.07 ± 1.62	3.13 ± 2.28	2.94 ± 2.13	0.001	0.001	0.006
PRV-SC	D_2%_ (Gy)	6.71 ± 2.83	9.34 ± 2.27	9.51 ± 2.29	0.001	0.001	0.088
	D_5%_ (Gy)	5.49 ± 2.66	7.91 ± 2.48	7.97 ± 2.58	0.001	0.001	0.712
	D_mean_ (Gy)	2.26 ± 1.82	3.37 ± 2.52	3.18 ± 2.38	0.001	0.001	0.006
Left optic nerve	D_2%_ (Gy)	4.97 ± 4.97	5.49 ± 4.56	5.03 ± 4.54	0.020	0.977	0.057
Right optic nerve	D_2%_ (Gy)	4.40 ± 3.34	5.19 ± 3.65	4.90 ± 4.06	0.001	0.065	0.211
Optic chiasm	D_2%_ (Gy)	6.47 ± 5.17	7.73 ± 5.45	7.50 ± 6.48	0.003	0.069	0.140
Left lens	D_2%_ (Gy)	2.11 ± 1.40	2.39 ± 1.39	2.25 ± 1.46	0.007	0.140	0.090
Right lens	D_2%_ (Gy)	1.98 ± 1.19	2.19 ± 1.31	2.19 ± 1.40	0.029	0.096	0.394
Left T-M joint	D_2%_ (Gy)	29.89 ± 9.14	30.29 ± 9.89	31.07 ± 10.14	0.733	0.173	0.650
Right T-M joint	D_2%_ (Gy)	31.68 ± 14.45	30.09 ± 14.66	32.43 ± 14.63	0.609	0.334	0.009
Oral cavity	D_mean_ (Gy)	16.47 ± 9.44	16.15 ± 8.77	15.87 ± 8.66	0.910	0.650	0.691
Left parotid	D_mean_ (Gy)	23.38 ± 12.33	23.99 ± 13.15	24.29 ± 13.22	0.776	0.650	0.191
Right parotid	D_mean_ (Gy)	20.76 ± 10.01	21.24 ± 10.04	22.50 ± 10.35	0.609	0.053	0.061
Left temporal lobe	D_mean_ (Gy)	13.95 ± 10.51	14.42 ± 10.35	14.28 ± 10.71	0.078	0.281	0.363
Right temporal lobe	D_mean_ (Gy)	11.43 ± 7.93	12.28 ± 8.51	11.98 ± 8.39	0.021	0.363	0.256
Normal tissue	D_mean_ (Gy)	5.81 ± 2.62	5.97 ± 2.69	5.89 ± 2.69	0.002	0.036	0.125
Monitor unit		965 ± 160	693 ± 95	715 ± 100	0.001	0.001	0.053

*Abbreviations:* V_100%_ = % volume covered by 100% of the prescription dose; HI = homogeneity index; CI = conformity index; other abbreviations as in Table 1.

**Table 3:** Linear-regression analysis of the relationship between the geometric parameters of the cases and the dose reductions of the (PRVs of) BS/SC by IS-RA compared to C-RA/RA-SGC.

		***P*****-value**					
		**IS-RA vs. C-RA**			**IS-RA vs. RA-SGC**		
		**Ang_BSSC**	**Dist_Min**	**Vol_PTV**	**Ang_BSSC**	**Dist_Min**	**Vol_PTV**
BS	D_2%_	0.054	0.090	0.108	0.745	0.596	0.986
	D_5%_	0.448	0.519	0.510	0.182	0.128	0.459
	D_mean_	0.018	0.032	0.063	0.008	0.007	0.102
PRV-BS	D_2%_	0.091	0.207	0.171	0.411	0.704	0.350
	D_5%_	0.144	0.276	0.184	0.712	0.941	0.509
	D_mean_	0.033	0.043	0.080	0.021	0.010	0.160
SC	D_2%_	0.197	0.073	0.217	0.491	0.407	0.663
	D_5%_	0.291	0.045	0.355	0.880	0.387	0.971
	D_mean_	0.868	0.177	0.661	0.943	0.286	0.955
PRV-SC	D_2%_	0.018	0.059	0.055	0.022	0.072	0.062
	D_5%_	0.030	0.021	0.076	0.180	0.083	0.273
	D_mean_	0.873	0.183	0.598	0.930	0.324	0.850

*Abbreviations:* Ang_BSSC = the angle extended by the PTV with respect to the axis of BS/SC; Dist_Min = the minimum distance between PTV and BS/SC; Vol_PTV = the volume of PTV; other abbreviations as in Tables 1 and 2.
